# An inguinal hernia ‘hard to stomach’

**DOI:** 10.1093/jscr/rjad416

**Published:** 2023-07-29

**Authors:** Shawn Liechty, Andrew D Eiref, Keerthivasan Vengatesan, Samuel P Barasch, Xiang D Dong, Patrick W Zimmerman, Michael Nicoara, Krishan Patel, Heath Walden, Simon D Eiref

**Affiliations:** Department of Surgery, Danbury Hospital, 24 Hospital Ave, Danbury, CT 06810, USA; Department of Surgery, Danbury Hospital, 24 Hospital Ave, Danbury, CT 06810, USA; Department of Surgery, Danbury Hospital, 24 Hospital Ave, Danbury, CT 06810, USA; Department of Pathology, Danbury Hospital, 24 Hospital Ave, Danbury, CT 06810, USA; Department of Surgery, Danbury Hospital, 24 Hospital Ave, Danbury, CT 06810, USA; Department of Surgery, Danbury Hospital, 24 Hospital Ave, Danbury, CT 06810, USA; Department of Surgery, Danbury Hospital, 24 Hospital Ave, Danbury, CT 06810, USA; Department of Surgery, Danbury Hospital, 24 Hospital Ave, Danbury, CT 06810, USA; Department of Surgery, Danbury Hospital, 24 Hospital Ave, Danbury, CT 06810, USA; Department of Surgery, Danbury Hospital, 24 Hospital Ave, Danbury, CT 06810, USA

## Abstract

Inguinal hernias containing the stomach are extremely rare, and have never been described in females. We are reporting the case of a 79 year old female who presented in septic shock with a left inguinal hernia containing the stomach, resulting in gastric perforation and loss of abdominal domain. She underwent emergency exploratory laparotomy with manual reduction of the hernia, wedge resection of the perforated gastric segment, abdominal washout, and closure of the abdominal fascia using biological mesh. She had a complicated but successful postoperative course, and was discharged to a rehabilitation center three weeks after hospital admission.

## INTRODUCTION

Inguinal hernias are commonly encountered in general surgery, accounting for 75% of all abdominal wall hernias, and undergo operative repair 1 million times a year in the United States. Inguinal hernias occur more commonly in males than females, with a lifetime risk of 27% and 3%, respectively [1]. Inguinal hernias frequently contain adjacent mobile structures, for example small or large intestine or omentum. However, inguinal hernias rarely contain distant fixed structures, such as the stomach; in fact, review of the literature from 1942–2020 revealed only 20 case reports of stomach-containing inguinal hernias. All cases were in males. Most were in the elderly, left-sided, and associated with either gastric outlet obstruction or gastric perforation [2]. These rare hernias likely result when attached omentum and colon become incarcerated, pulling the stomach down and into the hernia sac, slowly over time [3]. Operative management can be complex depending on whether there is a large hernia or concomitant gastric perforation [4]. Here, we describe the presentation and management of the first documented case of a stomach-containing inguinal hernia in a female, resulting in gastric perforation and loss of abdominal domain.

## CASE REPORT

**Figure 1:**
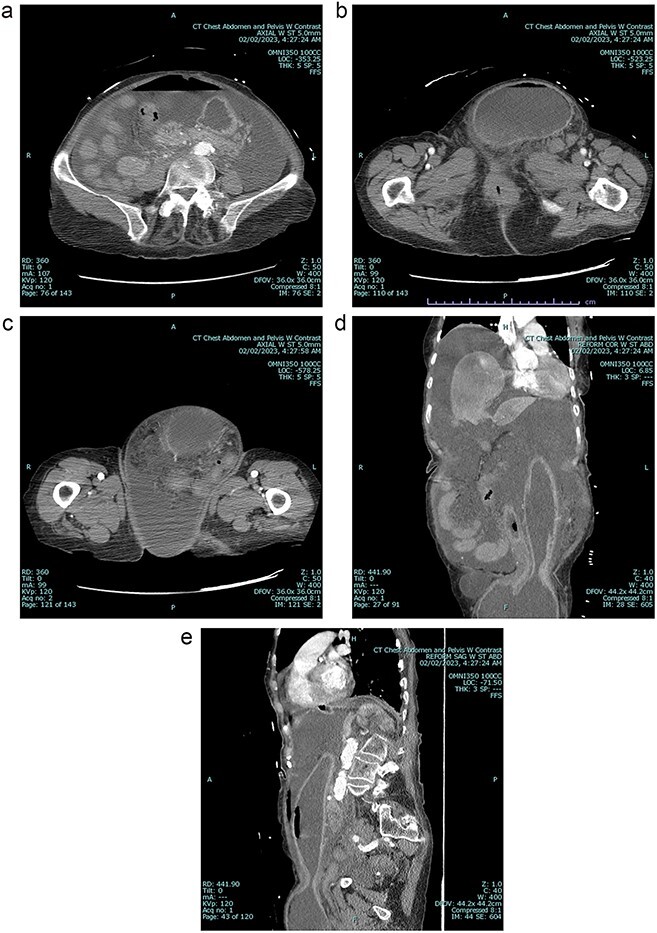
A Preoperative axial view at level of mid abdomen, demonstrating free intraabdominal fluid and air 1b: Preoperative axial view at level of groin, demonstrating stomach-containing left inguinal hernia 1c: Preoperative axial view at level of the mid thigh, demonstrating left inguinal hernia containing both the stomach and other viscera 1d: Preoperative coronal view, demonstrating stomach being pulled down into inguinal hernia sac 1e: Preoperative sagittal view, demonstrating stomach being pulled down into inguinal hernia sac, and associated free intraabdominal air.

**Figure 2:**
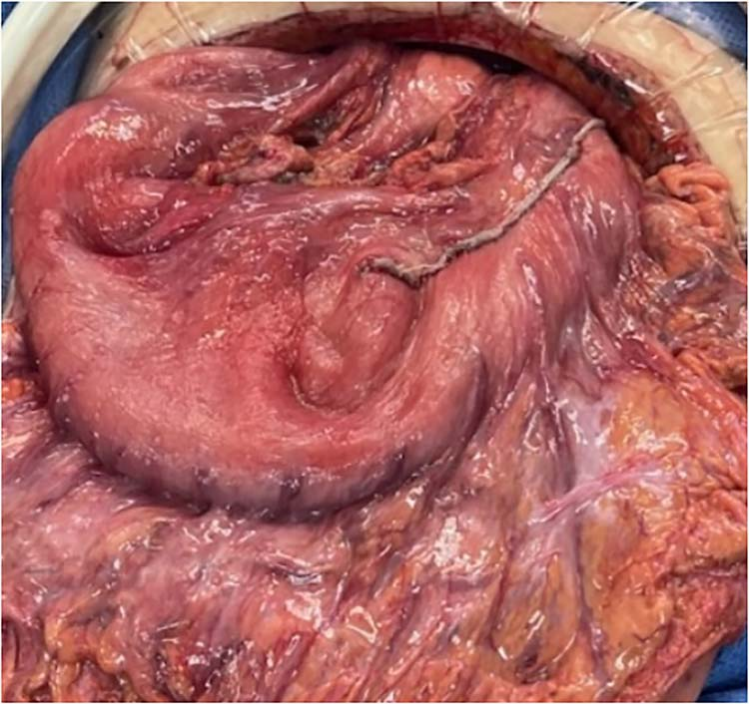
Intraoperative photo of stomach following gastric repair.

**Figure 3:**
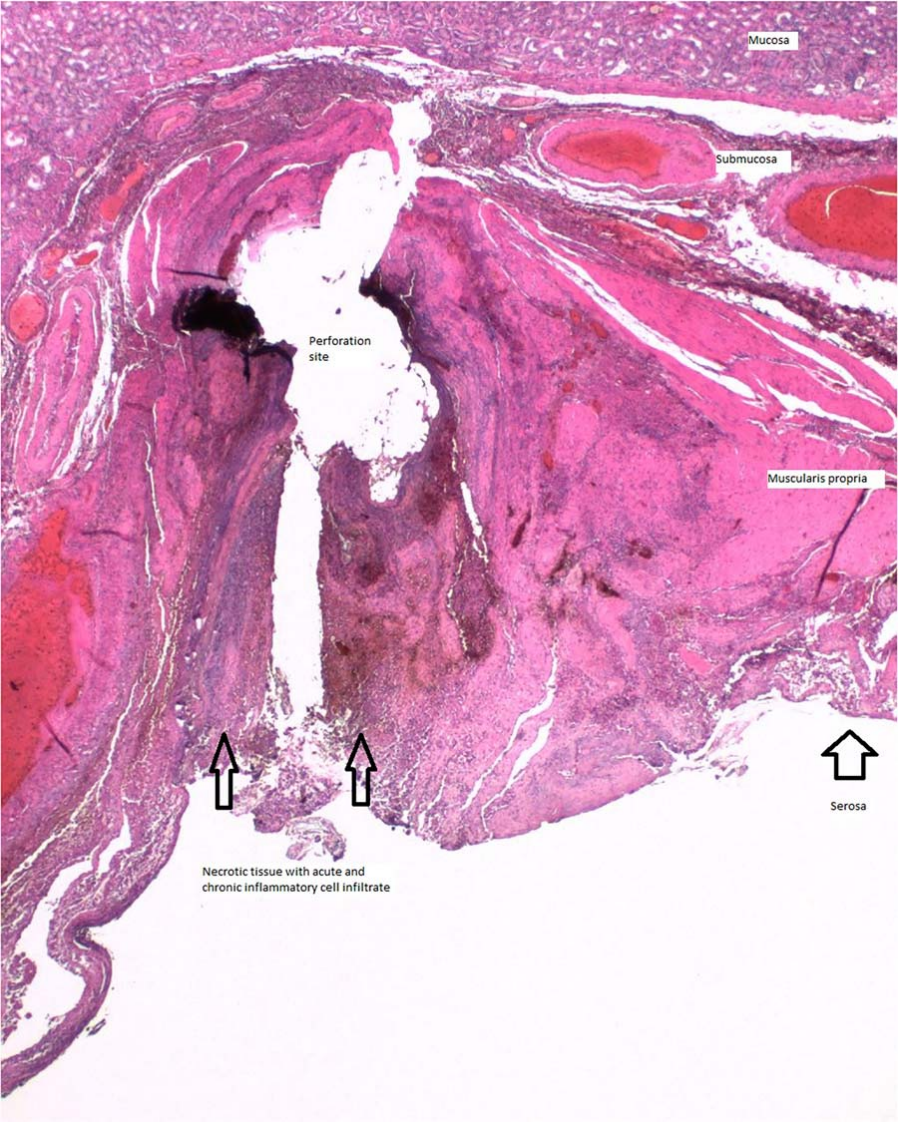
Histologic section of gastric perforation site with H&E stain, suggesting a pressure-related injury.

**Figure 4:**
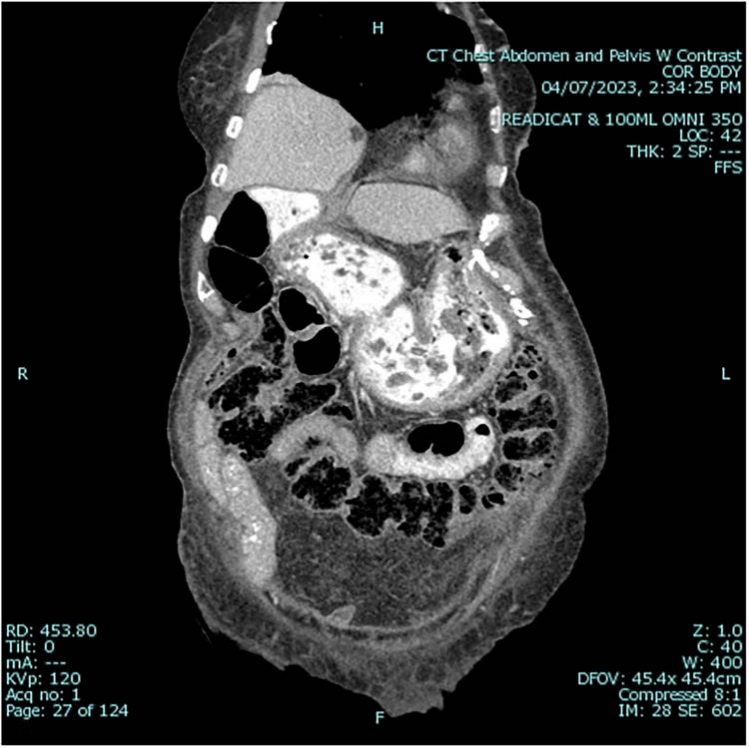
Postoperative sagittal view, demonstrating stomach back in normal anatomical position1.

A 79 year old female patient presented to the emergency department with a two-day history of intractable nausea, vomiting, and diffuse abdominal pain. She reported having had a left groin bulge for 70 years and did not regularly see a doctor. Vital signs were notable for tachycardia and tachypnea. Physical exam was notable for a firm and distended diffusely-tender abdomen; and an associated giant incarcerated left inguinal hernia, extending down to the thigh. Laboratory evaluation revealed leukocytosis (WBC 20 K/uL), lactic acidosis (lactate 5 mmol/L), and acute kidney injury (Cr 1.5 mg/d). Computed tomography imaging demonstrated a giant left inguinal hernia containing the stomach, proximal duodenum, right hemicolon, and omentum, and associated free intraabdominal fluid and pneumoperitoneum (Fig 1). Aggressive intravenous fluid resuscitation, broad spectrum antibiotics, and vasopressor-support were started for septic shock secondary to perforated viscus. The patient was taken emergently to the OR for exploratory laparotomy. Operative findings included the following: left inguinal hernia containing the aforementioned structures, herniation of the stomach with severe gastric distension, a 0.5 cm diameter gastric perforation with surrounding necrosis at the mid portion of the lesser curve, and large volume ascites containing gastric contents. Operative interventions included the following: manual reduction of bowel and omentum from the left inguinal hernia sac, wedge resection of the perforated stomach (Figs 2 and 3), and abdominal washout. The patient was noted to have loss of abdominal domain due to the longstanding hernia. Her abdomen was closed using a biological mesh in a bridging fashion, as the fascia could not be closed primarily. Wide drainage was achieved using four 19Fr Blake drains. After surgery, the patient recovered from septic shock while being cared for in the intensive care unit. Her postoperative course was complicated by atrial fibrillation, pulmonary embolism, and COVID-19 infection. Discharge to a rehabilitation center occurred on hospital day 20. Follow up computed tomography imaging is as shown (Fig 4). Staged repair of the left inguinal hernia with permanent prosthetic mesh is planned in the near future.

## DISCUSSION

This is the first reported case of a stomach-containing inguinal hernia in a female. Inguinal hernias containing the stomach are rare and medically challenging. They are typically large in size and may be complicated by perforation or loss of domain. Their surgical management involves the following steps: care of associated critical-illness, repair of gastric perforation, closure of the abdomen when there is loss of domain, and, finally, inguinal hernia repair.

Patients with stomach-containing inguinal hernias may present acutely and critically ill. Perforation of the stomach can lead to septic shock. Gastric outlet obstruction can lead to volume depletion and concomitant hypovolemic shock. Fluid resuscitation, early antibiotic administration, and source control are key upfront therapies [5]. Intensive care unit management may also be necessary during the perioperative period.

Patients with stomach-containing inguinal hernias may go on to develop acute gastric perforation, likely due to a combination of factors: pressure-related injury from gastric distension [6, 7], tearing of the stomach wall as it is pulled down into the hernia sac [4], and devascularization from possible incarceration and strangulation. Pressure-related injuries resulting in gastric perforation may be associated with ischemia and necrosis [6, 7] and require larger areas of resection; whereas, tears can sometimes be repaired primarily [8]. The approach used for gastric repair should be tailored to the size, perfusion, and location of the injury.

Patients with stomach-containing inguinal hernias may develop loss of domain, thereby making closure of the abdomen more complicated [2]. Giant hernias may house significant portions of the abdominal viscera over a prolonged period. Attempted reduction of this viscera back into the abdominal cavity can result in intra-abdominal hypertension and possible abdominal compartment syndrome, and prevent primary closure of the abdominal fascia. In this scenario, techniques such as visceral debulking, abdominal wall reconstruction, or mesh bridging can be used to facilitate abdominal closure. Preoperative progressive pneumoperitoneum to increase the volume of the abdominal cavity has also been described in the elective setting [9].

Patients with stomach-containing inguinal hernias may benefit from a staged repair of their inguinal hernia depending on the degree of contamination or hemodynamic instability [8]. Inguinal hernia repair is best performed with permanent prosthetic mesh in order to prevent recurrence [1]. However, current guidelines recommend against the use of permanent prosthetic mesh in dirty-contaminated surgical fields due to the high risk of mesh infection [10]. Consideration in cases where there is gross enteric spillage should be given to delayed mesh repair, following resolution of abdominal sepsis and medical optimization. A staged approach here can help to reduce morbidity and mortality. We hope that our findings may be of use to other surgeons dealing with this rare condition.

## Data Availability

All data underlying the results are available as part of the article and no additional source data are required.
